# 

**Published:** 1882-10-20

**Authors:** 


### TABLE OF CONTENTS.

ORIGINAL AND SELECTED ARTICLES.

Veratrum Viride in Pureperal Convulsions, by R. F. McConnell, M.D., of Alabama, 261. Replacing and Healing of Pieces Separated from the Human Body, by G. Halstead Boyland, M. D. M.A., of Baltimore, 364. Retention of Placenta, by J. V. White, M. D., C.M., M.C.P. & S., Ont., Au Sable, 366. Tincture of Perchloride of Iron in Post Partum Hemorrhage, by J. H. Scarff, M.D., of Baltimore, 368. Palcenta Prævia, by J. D. Strawbridge, M.D., of Danville, Penn., 370 A New Method of Treating Uterine Hemorrhage, by M. R. Barker, M.D., of New Castle, Penn., 373. The Evil Effects of the Nasal Douche, by Carl Seiler, M.D., 374. How to Examine a Sick Child, translated by R. Matas, M.D., 376. The Bacillus of Tuberculosis, 377.

ABSTRACTS AND GLEANINGS.

Foreign Bodies in the Air Passages; The Rational and Routine Treatment of Venereal Diseases, 378. Value of Pure Codine, 379. Can a Man have Syphilis Twice? 380. Physiological Effects of Prolonged Bathing, 381. Shall the Traveling Mesmerizer be Abolished? 382. Treatment of Bone Felon, 383. Case of Pregnancy at the Age of Sixty-two, 384 The Scientific Principles of Inhalation, 385 Tonsillotomy by Ignipuncture; Swallowing a Baby, 386. The Feeding of Infants; Can Dreams be Controlled?; Treatment of Facial Erysipelas by Scarifications and Opium, 387. Recent Advances in Urinaey Analysis; Simple Treatment of Excessive Sweating; Comparative Size of Drops, 388. Codeia in Diabetes; Hydrophobia Cured; Detection of Vesical Calculi in Children; Hydrate of Chloral and Tincture of Iodine; An Irish Hermaphrodite, 389. Disinfection of Urine; Ataxy and Sewing Machines; American Medical Association; A New Cinchona Alkaloid, 390.

SCIENTIFIC ITEMS.

Curious Fact Concerning Boiling Water; Mica Masks, 391. The Center of Population; A Curious Bronze Coin; Dynamogen; Materialists, 392.

PRACTICAL NOTES AND FORMULÆ.

For Tonsilitis; Iodine in Croupal Pneumonia; To Remove Freckles; Chandler's Chlorodyne, 393. Mistura Opii Composita; A Sedative Emmenagogue; A New Remeey for Tape-Worm; Salicylic Cream; Granular Eyelids, 394. Mosquito Oil; Membranous Dysmenorrhea; Cholera Pills, Neuralgia; Flea Bites, etc., 395.

EDITORIALS AND MISCELLANEOUS.

Local Notices; Embalming Match; Encouraging to Women Doctors; Epidemic Uticaria, 396. Quinine Flower; City and Country; Mississippi State Medical Society, 397. Sanitary Convention; Arkansas State Medical Society, 398. Book Notices, 399. Special Notices 400.

